# Retraction: Unlocking Early Cancer Detection: Exploring Biomarkers, Circulating DNA, and Innovative Technological Approaches

**DOI:** 10.7759/cureus.r203

**Published:** 2025-11-19

**Authors:** B. Krishna Prasanth, Saad Alkhowaiter, Gaurav Sawarkar, B. Divya Dharshini, Ajay R. Baskaran

**Affiliations:** 1 Department of Community Medicine, Sree Balaji Medical College and Hospital, Bharath Institute of Higher Education and Research, Chennai, IND; 2 Department of Gastroenterology, College of Medicine, King Khalid University Hospital, Riyadh, SAU; 3 Rachana Sharir, Mahatma Gandhi Ayurveda College, Hospital and Research Centre, Datta Meghe Institute of Higher Education and Research, Wardha, IND; 4 Department of Biochemistry, Government Medical College, Khammam, Telangana, IND; 5 Department of Psychiatry, National Health Service, Shrewsbury, GBR

The Editors-in-Chief have retracted this article. An investigation by the journal found evidence of authorship manipulation. The Editors-in-Chief therefore no longer have confidence in the provenance and reliability of the article contents. The authors disagree with the decision to retract.

